# Comprehensive analysis and experimental validation reveal elevated CLCN4 is a promising biomarker in endometrial cancer

**DOI:** 10.18632/aging.204994

**Published:** 2023-09-05

**Authors:** Chenyang Wang, Jing Li, Weina Liu, Shiya Li, Yi Zhang, Yanbin Jin, Jinquan Cui

**Affiliations:** 1 Department of Gynecology, The Second Affiliated Hospital of Zhengzhou University, Zhengzhou 450000, China; 2 Academy of Medical Sciences, Zhengzhou University, Zhengzhou 450000, China; 3 Department of Gynecology, Qingdao Hospital, University of Health and Rehabilitation Sciences (Qingdao Municipal Hospital), Qingdao, Shandong 266000, China; 4 Department of Gynecology, The University of Auckland, Grafton, Auckland 1023, New Zealand; 5 Department of Gynecology, Hainan Affiliated Hospital of Hainan Medical University (Hainan General Hospital), Haikou 570311, China

**Keywords:** CLCN4, endometrial cancer, biomarker, prognosis, CD4+ T-cell

## Abstract

Several studies have reported the role of CLCN4 in tumor progression. However, its mechanism remains to be thoroughly studied. The objective of this study was to explore the potential pathogenic role of CLCN4 in endometrial carcinoma (UCEC) with a better understanding of the pathological mechanisms involved. The potential roles of CLCN4 in different tumors were explored based on The Cancer Genome Atlas (TCGA), the expression difference, mutation, survival, pathological stage, Immunity subtypes, Immune infiltration, tumor microenvironment (TME), tumor mutation burden (TMB), microsatellite instability (MSI), mismatch repair (MMR) related to CLCN4 were analyzed. Then, the expression, prognosis, mutation, and functional enrichment of CLCN4 in UCEC were analyzed. Immunohistochemical experiment was used to verify the expression of CLCN4 in endometrial cancer tissues and normal tissues. *In vitro*, we knocked down of CLCN4 in HEC-1-A cells and performed CCK8, WB, RT-PCR, wound-healing, transwell assays to further validation of the molecular function. Results revealed that high expression of CLCN4 was observed in 20 cancer types of TCGA. CLCN4 expression correlates with poor survival in MESO, BLCA, THCA, especially UCEC tumors. CLCN4 expression was significantly associated with CD4+ T-cell infiltration, especially CD4+ Th1-cell. Immunohistochemical experiment reveals that CLCN4 is high expressed in endometrial tumors, *in vitro* experiment reveals that knockdown of CLCN4 inhibits the cells proliferation, migration and invasion. Our study is the first to offer a comprehensive understanding of the oncogenic roles of CLCN4 on different tumors. CLCN4 may become a potential biomarker in UCEC.

## INTRODUCTION

In terms of gynecologic cancers, endometrial carcinoma of the uterus corpus (UCEC) is the second most common type [1], and its incidence has steadily increased over the past decades [2, 3]. A major reason for this is the increase in obesity rates and diabetes, as well as the aging of the population and the decline in use of combined menopausal hormones [4, 5]. UCEC is traditionally classified into two types. The majority are low-grade endometrioid adenocarcinoma, which are estrogen-driven and tend to a good prognosis [6]. Tumors of type II include serous, clear-cell, carcinosarcoma, and undifferentiated carcinomas, despite representing only 15–20% of all UCEC cases, these tumors account for over 50% of all deaths because of their propensity for metastases and resistance to chemotherapy [7]. Screening for endometrial cancer is therefore of great importance. In most cases, UCEC is diagnosed through fractional curettage or endometrial biopsy during hysteroscopy [8], which is a complex operation that may lead to uterine perforation, infection, and pain [9]. There are currently no reliable biomarkers for the disease’s prognostic implications. In recent years, high-throughput platforms for gene expression have been widely used to predict prognoses [10, 11], identify new drugs [12], and classify patients [13, 14]. Therefore, understanding the key genes involved in tumor progression and their clinical prognosis is fundamental in exploring their underlying molecular mechanisms [15].

A chloride channel is believed to maintain cellular membrane potential, cellular volume, electrostatic compensation, as well as keeping the pH of lysosomes or intracellular organelles. Additionally, chloride movement regulate cancer cell motility and metastasis. In mammals, CL gene family contains nine members, four (ClC-1, ClC-2, ClC-Ka and ClC-Kb) encode plasma membrane chloride channels, while five (ClC-3–7) encode chloride exchangers intracellularly [16, 17]. The CLCN4 gene on Xp22.2 of the human chromosome, which codes for ClC-4, is a voltage-dependent chloride-hydrogen ion exchanger [18–20]. CLCN4 is expressed extensively in the brain, heart, liver, kidney, and intestine [21, 22]. In 1999, Prof. Soroceanu made the first assertion that inhibiting chloride channels with drugs reduced the movement and infiltration of glioma cells in fetal rat brain tissue [23]. However, this investigation was not identified that which chloride channel drive migration or invasion, for at least 11 separate exchangers having been reported [24, 25]. In 2010, Dr. T. Ishiguro identified CLCN4 as a novel promoter of colon cancer migration, invasion, and metastasis [26]. It is unknown whether CLCN4 plays a role in other types of tumors.

Using the public database TCGA, we analyzed the pathogenic genes of each tumor, and found a significant correlation between CLCN4 gene expression and survival, immune infiltration, and genetic instability, particularly in UCEC. Then, a comprehensive analysis was conducted on CLCN4 to investigate its pathogenic mechanism in UCEC, and cell experiments confirmed that it weakens tumor proliferation, migration and invasion. There are some signs that CLCN4 may be related to the evolution of endometrial cancer, according to the findings.

## MATERIALS AND METHODS

### Genome-wide expression analysis

We downloaded clinical and phenotypic information, somatic mutation data, and RNA sequencing data from the TCGA database (https://portal.gdc.cancer.gov/). Downloads of the gene expression data for normal tissues were made using the Genotype-Tissue Expression database (https://portals.broadinstitute.org/ccle/). Data were downloaded from the CCLE database (https://sites.broadinstitute.org/ccle/) for each tumor cell line. Using the input “CLCN4”, the tumor immunity estimation resource, web server version 2 (TIMER2) (http://timer.cistrome.org/), was examined. We transformed the expression data to Log2 and ran two serial *t*-tests to see if there was a difference in expression between tumor and normal tissue. It was deemed significant at a P^*^0.05. R software (version 4.1.0; http://www.Rproject.org) and the DESeq2-based R pipeline for systematically differential analysis of RNA-Seq data were used for our data analysis. A UALCAN database (http://ualcan.path.uab.edu/) correlates CLCN4 expression with tumor stage.

### Analyzing the survival prognosis

In order to determine the survival outcomes between the high- and low-expression groups of CLCN4, we performed a Kaplan-Meier survival analysis. Based on univariate Cox regression models, overall survival (OS), disease-specific survival (DSS), disease-free survival (DFS), progression-free survival (PFS) determined. KM analysis was performed using R packages “survminer” and “survival”, while forest plots of Cox regression used “survival” and “forestplot” to display *p*-values, HRs, and 95% CIs. Based on a nomogram developed using the “rms” package, rates of overall recurrence were predicted for 1, 3, and 5 years.

### Correlation with clinical stages

By analyzing each TCGA sample, CLCN4 expression was assessed for association with tumor stages, which was visualized using “ggplot2”.

### The value of CLCN4 analyzed by TISIDB

Tumor immunoassays can be carried out using the TISIDB platform (http://cis.hku.hk/TISIDB/index.php), which contains a large amount of heterogeneous data sets. Using TISIDB data, we investigated the relationship between CLCN4 expression and immune and molecular subtypes. We used box-plots to illustrate and display the results.

### Correlation analysis of CLCN4 expression in tumor microenvironment

Tumor microenvironments (TME) are the conditions under which cancer cells grow and survive. A tumor’s constituents include stromal cells, immune cells surrounding the tumor, and tumor cells themselves. ESTIMATE-Score, Immune-Score, and Stromal-Score were all computed with R’s “ESTIMATE” package. Then we used R’s Spearman correlation analysis to determine if CLCN4 is related to both stromal and immunological scores.

### Analysis of immune infiltration

22 different immune cell subtypes were used to measure tumor purity using the “immune Genes” module of the TIMER2 web server. With the aid of the TIMER, CIBERSORT, CIBERSORT-abs, QUANTISEQ, XCELL, MCPCOUNTER, and EPIC algorithms, the immune infiltration was calculated. Rank-sum tests were used to detect differences between two groups. An acceptable *P*-value was 0.05.

### CLCN4 expression with MSI and TMB correlation

The tumor mutation burden (TMB) is a biomarker that indicates a mutation in cancer. Using Spearman’s rank correlation coefficient, TMB was computed independently for each tumor sample. The microsatellite instability (MSI) is caused by repeating units being added or deleted, resulting in a longer or shorter microsatellite than normal. The “reshape2” and “RColorBrewer” R-pack packages were used to build bubble charts that displayed the results.

### Correlation analysis between CLCN4 and some genes

Histone modifications known as DNA methylation affect gene expression. Mismatch Repair Genes (MMRs) play a critical role in the intracellular mismatch repair mechanism [27]. The use of immunostimulatory antibodies directed toward immune receptor molecules is an exciting new approach in cancer treatment. Immunostimulants work by antagonizing receptors that suppress immune responses or activating others that increase immune responses [28]. In the fight against cancer, copper may pose a vulnerability since it plays a crucial role in its genesis, severity, and course. As shown by heat maps, using “RColorBrewer”, we plotted the relationships between CLCN4 expression, MMRs, immune stimulants, and copper death-related genes.

### Enrichment analysis

Functional enrichment was used to confirm that prospective targets might have functional properties. Ontologies (GO) are popular techniques for classifying gene functions, particularly cellular components (CC), biological pathways (BP), and molecular functions (MF). The KEGG enrichment analysis provides information on gene functions, as well as high-level genomic functionality. “GOplot” and “cluster profiler” packages were used to examine GO function and to KEGG pathways to better understand target gene-carcinogenesis.

### Immunohistochemistry

For immunohistochemistry, 10 carcinoma of endometrium sections were stripped. The UCEC tissue sections were subjected to deparaffinization and rehydration. The expression of CLCN4 was determined by staining with polyclonal rabbit anti-mouse CLCN4 antibody overnight at 4°C. As controls, 10 irrelevant normal endometrial tissue were selected. After phosphate buffered saline rinsing, sections were treated with an anti-CLCN4 antibody (Abcam ab75008, 1:1000) and incubated overnight at 4°C. After being stained, counterstained, dried off, all sections were detected using the peroxidase and anti-peroxidase method. A yellow particle in the cytoplasm or nucleus was utilized to estimate the percentage of endometrial cancer cells. Staining strength was graded as negative (–), weakly positive (+), medium (+++), or very positive (+++). H-scores are calculated by multiplying the intensity score by the proportion of positive cells, which ranges from 0 to 300. H-scores were computed by two experienced pathologists using a double-blind process.

### Experimental validation of CLCN4 *in vitro*

#### Cell culture

Endometrial cancer cell line HEC-1-A was purchased from China Type Culture Collection (Procell, Wuhan), The Gibco company provided DMEM High Glucose culture medium, and with 10% fetal bovine serum (FBS), as well as 100 U/ml penicillin-streptomycin combination. (P/S).

### Knockdown of CLCN4

We have developed recombinant lentiviral vectors for CLCN4 RNAi (LV-CLCN4-RNAi) and for negative control (LV-Ctrl) in 293T cells manufactured by Gene Chem Co., Ltd. The siRNA sequence was as follows: 5′-GGCUGAUGUUUGUAACUUA-3′. The sh-CLCN4/sh-Ctrl cohorts had HEC-1-A cells (1 × 10^4^/well) sub-cultured in 24-well culture plates and infected with LV-CLCN4-RNAi/LV-Ctrl for 16 h. Then incubating the infected cells with puromycin 2 μg/mL for 48 hours, the infected cells were selected. The knockdown efficiency of CLCN4 was determined by Western blotting and RT-PCR.

### RNA extraction and quantitative real-time PCR

TRIzol reagent (Thermo Fisher Scientific, 15596026) was used to extract RNA from HEC-1-A cells. A NanoDrop 2000 (Thermo Fisher Scientific, Inc.) instrument was used to determine RNA concentration. qPCR was performed using the ChamQ™ SYBR^®^ qPCR Master Mix (High ROX Premixed; Vazyme) based on the manufacturer’s instructions. The relative gene expression was calculated using the 2^−ΔΔCq^ method [29]. The primers were the following: β-actin forward, ATGATGATATCGCCGCGCT and reverse AGGATGCCTCTCTTGCTCTG, CLCN4 forward TGATCAGCTCAGCACTTCCA and reverse CATCCTCTCCACAGCCGTAT. Three repetitions of each experiment were performed.

### Western blotting

Total proteins (including from HEC-1-A cells of the NC group, control group, CLCN4 knockdown group) were extracted using RIPA lysis buffer (APPLYGEN)with Protease Inhibitor Cocktail (Proteintech). Sodium dodecyl sulfate (SDS)-PAGE was used to separate proteins and PVDF filter membranes were used to store the protein samples (Millipore, USA). Membranes were blocked in TBST with 0.05% Tween-20 and 5% non-fat milk powder, and then incubated with primary antibodies as follows: CLCN4 (Abcam ab75008, 1:500), beta-actin (Proteintech, 20536-1-AP, 1:4000), Secondary antibodies were Horseradish peroxidase (HRP)-conjugated anti-Rabbit IgG (Fc) ((Proteintech, SA00001-2, 1:8000). Following visualization, the gray levels of the bands were quantified using ImageJ software.

### Cell proliferation assay

Cell viability assays were performed by counting and plating cells into 96-well plates (5000–6000 per well) 24 hours after stable cell lines (plasmid-con or knockdown). Cell viability were determined using Dojindo Corporation’s Cell Counting Kit-8 (CCK8) Assay Kit (Japan) as follows: The kit reagent is dissolved in DMEM Medium to prepare a 10% working solution after cell proliferation for 0/24/48/72 hours. After 2 h incubation in the 37°C incubator. To calculate the number of cells, the absorbance was measured at 450 nm three times.

### Wound-healing assay

For sh-CLCN4/sh-Ctrl group, HEC-1-A cells were grown in six-well plates (at a density of 4 × 10^4^ cells per cm^2^) for 24 hours. Then, 10 L sterile disposable pipettes were used to make scratches evenly. The samples were then rinsed twice with PBS and incubated in DMEM High Glucose medium containing 5% FBS. After scratching at the same position for 0 hours, 12 hours, and 24 hours, wound healing was examined. Three duplicate wells were tested.

### (Matrigel) migration assays

In the migration assays, 8-mm transwell chambers were used in 24-well plates (Costar). Trypsin (0.25%) was used to digest cells and then non-serum culture medium (DMEM) was added to resuspend the cells. We suspended 5000 cells in 300 liters of DMEM and seeded them for transwell chambers in the upper well after cell counting. For the matrigel transwell assay, the lower chamber was coated with matrigel matrix glue (Corning, serum-free medium: matrix glue = 8:1), and the cells were incubated at 37°C for 24 hours. Cells migration was stimulated in the well below the transwell chamber by adding 600 L of DMEM with 10% FBS. The transwell chambers were then removed from the 24-well plate and non-migrated cells were wiped off the membrane and the upper surface with cotton swabs after 24 hours. A 5% paraformaldehyde solution (Sinopharm Chemical Reagent Co, Ltd) was used to fix cells that migrated to the bottom of the chamber for 10 minutes, and hematoxylin-eosin (HE) staining was used to follow the cells. A minimum of five random fields were taken to image and count the membrane with cells. Three independent assays were conducted.

### Data and statistical analysis

This research was conducted using R software (version 4.1.0; https://www.r-project.org/). To examine differences between healthy and malignant tissue, the standard Wilcoxon’s test or Spearman’s test was applied. The overall survival rates of the various groups were compared using Kaplan-Meier analysis and log-rank testing. Subtypes, clinicopathological characteristics, risk scores, and immune infiltration were discovered using Pearson correlation, and the findings were deemed significant when *p* < 0.05.

### Data availability statement

The datasets presented in this study can be found in online repositories. The names of the repository/repositories and accession number(s) can be found in the Article/Supplementary Materials.

## RESULTS

### Data from gene expression analysis

**Figure 1:**
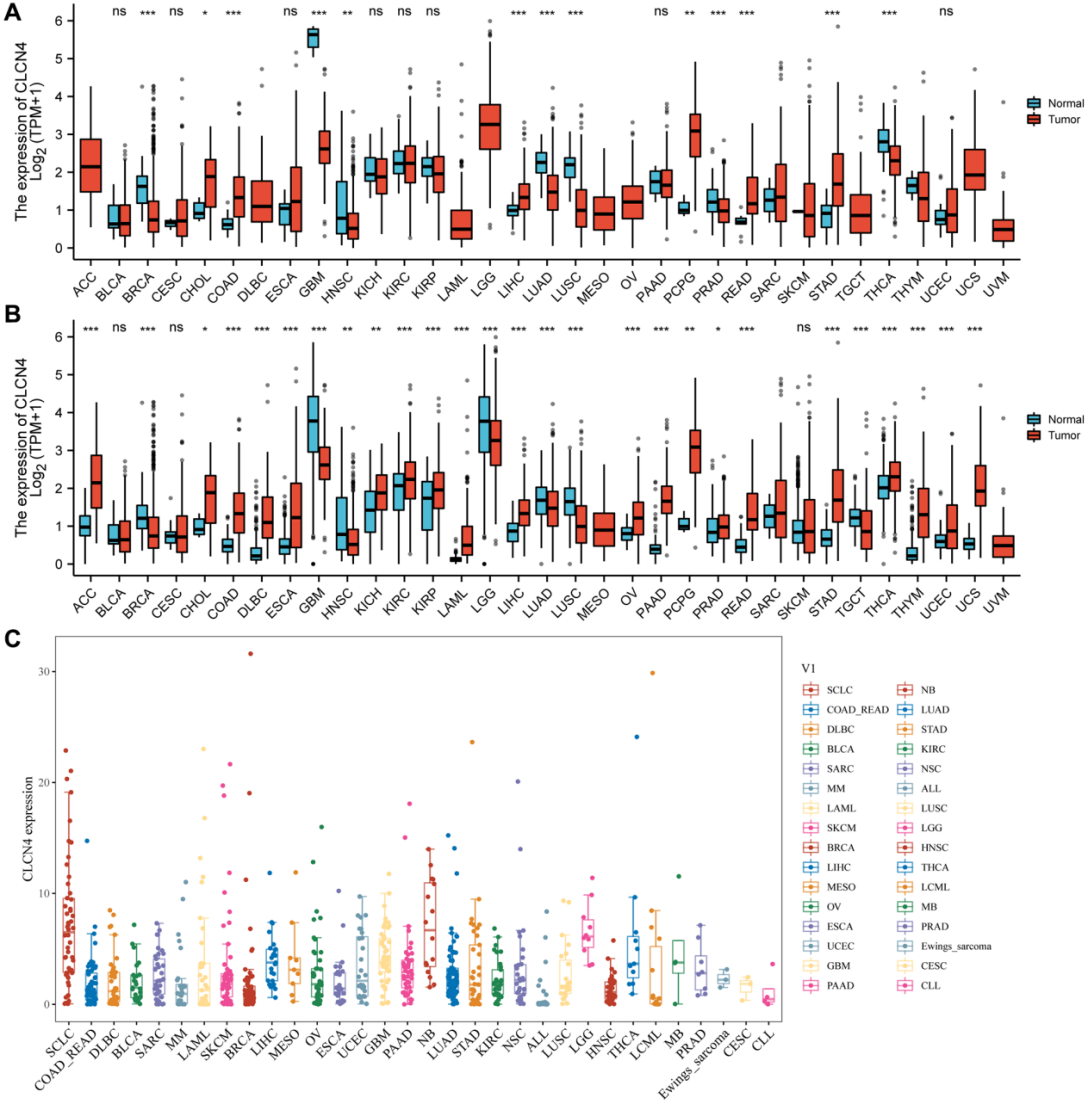
**The expression of CLCN4.** (**A**) Pan-cancer expression level of CLCN4 in TCGA dataset. (**B**) Pan-cancer expression levels of CLCN4 in TCGA and GTEx datasets. (**C**) The CLCN4 expression in various tumor cell lines in CCLE database. ^*^p < 0.05; ^**^p < 0.01; ^***^p < 0.001.

An analysis of the TCGA dataset revealed that CLCN4 expression is significantly increased in 6 cancer types, including cholangiocarcinoma (CHOL), colon adenocarcinoma (COAD), liver hepatocellular carcinoma (LIHC), pheochromocytoma and paraganglioma (PCPG), rectum adenocarcinoma (READ), and stomach adenocarcinoma (STAD) (*P* < 0.05), while it was significantly decreased in 6 tumors: breast invasive carcinoma (BRCA), glioblastoma multiforme (GBM), lung adenocarcinoma (LUAD), prostate adenocarcinoma (PRAD), lung squamous cell carcinoma (LUSC), thyroid carcinoma (THCA) tumor tissues (*P* < 0.05), and no significant difference was found in bladder urothelial carcinoma (BLCA), cervical squamous cell carcinoma and endocervical adenocarcinoma (CESC), esophageal carcinoma (ESCA), kidney chromophobe (KICH), kidney renal clear cell carcinoma (KIRC), kidney renal papillary cell carcinoma (KIRP), pancreatic adenocarcinoma (PAAD), uterine corpus endometrial carcinoma (UCEC) (Figure 1A).

Because normal tissue is significantly less abundant in the TCGA database than tumor tissue, we integrated the data from GTEx with TCGA (Figure 1B). CLCN4 expression was significantly different in 27 cancers. The majority of them were highly expressed, including adrenocortical carcinoma (ACC), CHOL, COAD, diffuse large B-cell lymphoma (DLBC), ESCA, kidney chromophobe (KICH), KIRC, KIRP, acute myeloid leukemia (LAML), liver hepatocellular carcinoma (LIHC), ovarian serous cystadenocarcinoma (OV), PAAD, PCPG, prostate adenocarcinoma (PRAD), READ, STAD, THCA, thymoma (THYM), UCEC, uterine carcinosarcoma (UCS) (*P* < 0.05). Additionally, low expression in BRCA, GBM, head and neck squamous cell carcinoma (HNSC), brain lower grade glioma (LGG), LUAD, lung squamous cell carcinoma (LUSC), testicular germ cell tumors (TGCT). The CCLE database was then used to demonstrate CLCN4 expression in various tumor cell lines (Figure 1C).

### Survival analyses

**Figure 2:**
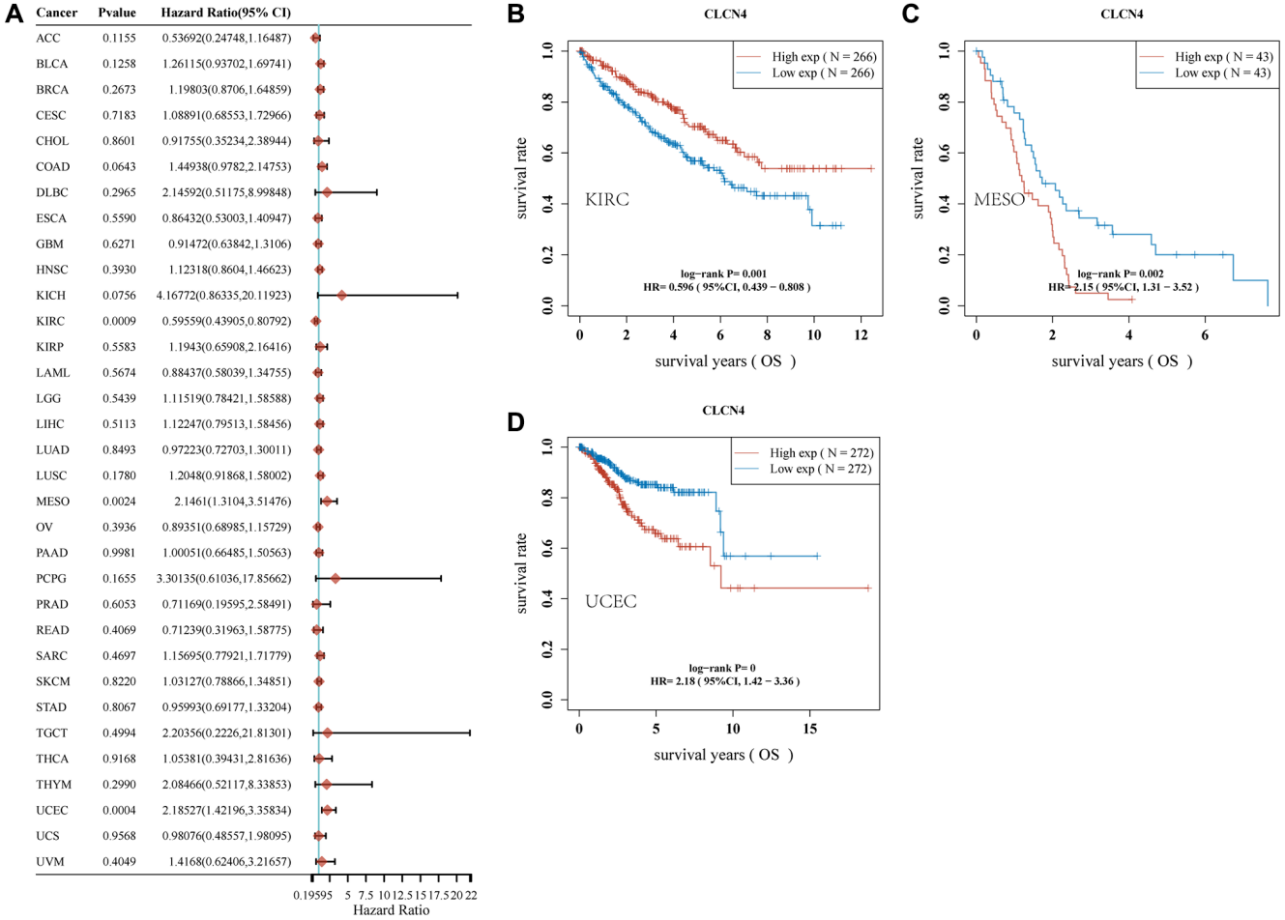
**Association of CLCN4 with OS in pan-cancer.** (**A**) The correlation between CLCN4 expression and OS in various tumors used cox regression model. (**B**–**D**) Kaplan-Meier curve of KIRC, MESO, UCEC.

We investigated if the prognosis, which comprises overall survival (OS), disease-specific survival (DSS), disease-free survival (DFS), and progression-free survival (PFS), was correlated with CLCN4 expression. Cancer cases were split into two groups based on the expression level of CLCN4, high expression, and low expression. According to Figure 2, malignancies like MESO (*p* = 0.002) and UCEC (*p* < 0.001) have a poor prognosis (OS) when CLCN4 is highly expressed. High CLCN4 expression is linked with the BLCA (*p* = 0.018), MESO (*p* = 0.021), and UCEC (*p* < 0.001), according to DSS analysis (Supplementary Figure 1). High CLCN4 expression is linked with BLCA (*p* = 0.0029) and UCEC (*p* < 0.001), according to PFS analysis (Supplementary Figure 2). High CLCN4 expression is linked with the THCA (*p* = 0.0036) and UCEC (*p* = 0.0016), according to DFS analysis (Supplementary Figure 3). However, the high expression of CLCN4 may be a protective factor for KIRC (OS *p* = 0.001, PFI *p* = 0, DSS *p* = 0). According to the aforementioned results, the prognosis of cases with some malignancies was connected with CLCN4 expression, especially in UCEC. Interestingly, across all four categories of prognostic factors, CLCN4 represents a risk factor for UCEC.

### Clinical correlation analysis

**Figure 3:**
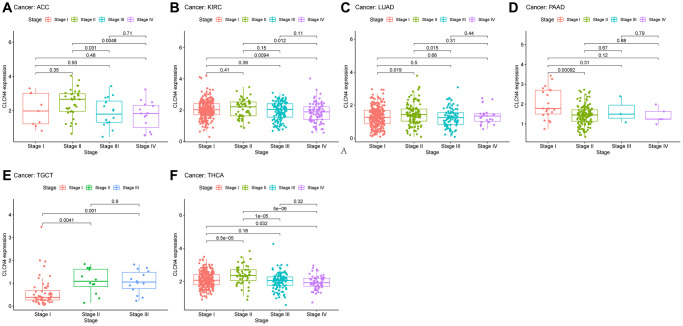
**The association between CLCN4 expression and cancer pathological grades (stage I, II, III, IV).** (**A**) ACC. (**B**) KIRC. (**C**) LUAD. (**D**) PAAD. (**E**) TGCT. (**F**) THCA.

According to the TCGA, CLCN4 expression increased with tumor stage in LUAD, TGCT, and THCA in stage II, and stage III increased in TGCT, however decreased in stage IV in KIRC. It suggests that CLCN4 may be useful for detecting malignancies clinically (Figure 3).

### Subtypes of immunity and molecules

**Figure 4:**
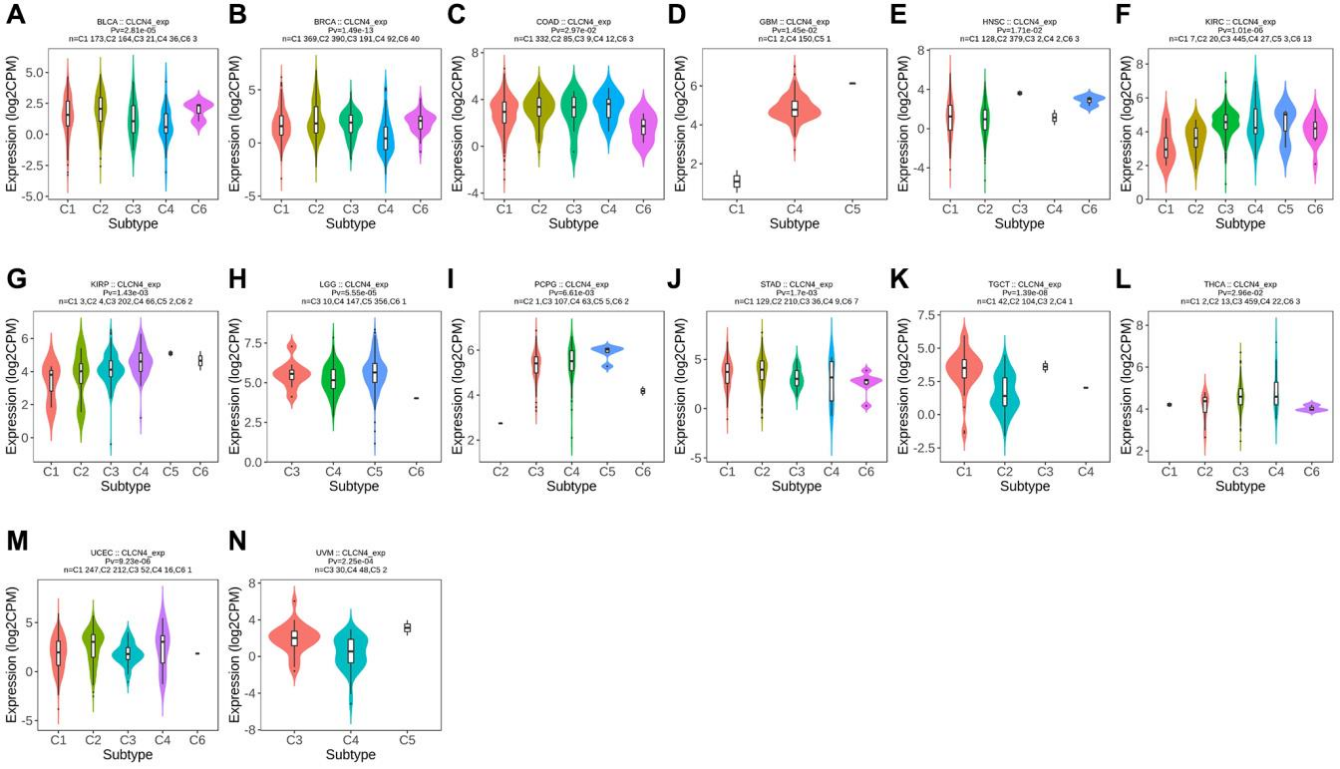
**Correlation between CLCN4 expression and tumor immune subtypes.** Expression of CLCN4 in TISIDB in different tumor immune subtypes. (**A**) BLCA. (**B**) BRCA. (**C**) COAD. (**D**) GBM. (**E**) HNSC. (**F**) KIRC. (**G**) KIRP. (**H**) LGG. (**I**) PCPG. (**J**) STAD. (**K**) TGCT. (**L**) THCA. (**M**) UCEC. (**N**) UVM.

**Figure 5:**
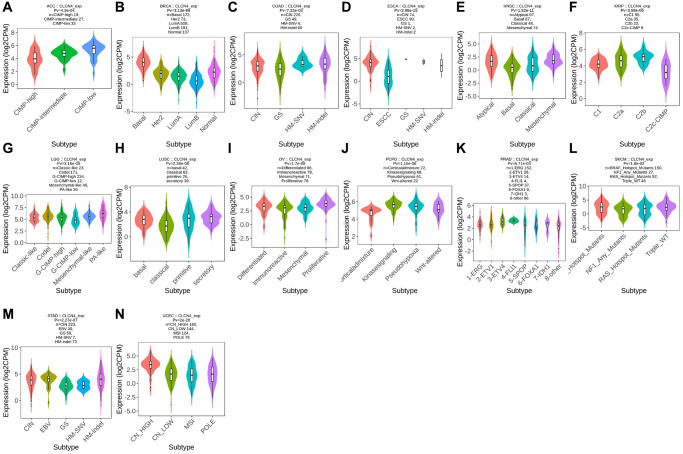
**Correlation between CLCN4 expression and tumor immune molecules.** Expression of CLCN4 in TISIDB in different tumor molecular subtypes. (**A**) ACC. (**B**) BRCA. (**C**) COAD. (**D**) ESCA. (**E**) HNSC. (**F**) KIRP. (**G**) LGG. (**H**) LUSC. (**I**) OV. (**J**) PCPG. (**K**) PRAD. (**L**) SKCM. (**M**) STAD. (**N**) UCEC.

Using the TISIDB database, we examined the connections between the expression of CLCN4 and immune subtypes and molecular subtypes. In immune subtype, CLCN4 expression was correlated with BLCA (*P* = 2.81E-05), BRCA (*P* = 1.49E-13), COAD (*P* = 2.97E-02), GBM (*P* = 1.45E-02), HNSC (*P* = 1.71E-02), KIRC (*P* = 1.01E-06), KIRP (*P* = 1.43E-03), LGG (*P* = 5.55E-05), PCPG (*P* = 6.61E-03), STAD (*P* = 1.7E-03), TGCT (*P* = 1.39E-08), THCA (*P* = 2.96E-02), UCEC (*P* = 9.23E-06), and UVM (*P* = 2.25E-04) (Figure 4). In molecular subtype, CLCN4 expression was correlated with ACC (*P* = 4.3E-04), BRCA (*P* = 3.13E-89), COAD (*P* = 7.22E-03), ESCA (*P* = 2.88E-15), HNSC (*P* = 1.52E-11), KIRP (*P* = 3.59E-06), LGG (*P* = 3.16E-05), LUSC (*P* = 2.38E-06), OV (*P* = 1.7E-08), PCPG (*P* = 1.15E-06), PRAD (*P* = 6.71E-03), SKCM (*P* = 1.8E-02), STAD (*P* = 2.27E-07), and UCEC (*P* = 2E-28) (Figure 5).

### Tumor microenvironment analysis data

**Figure 6:**
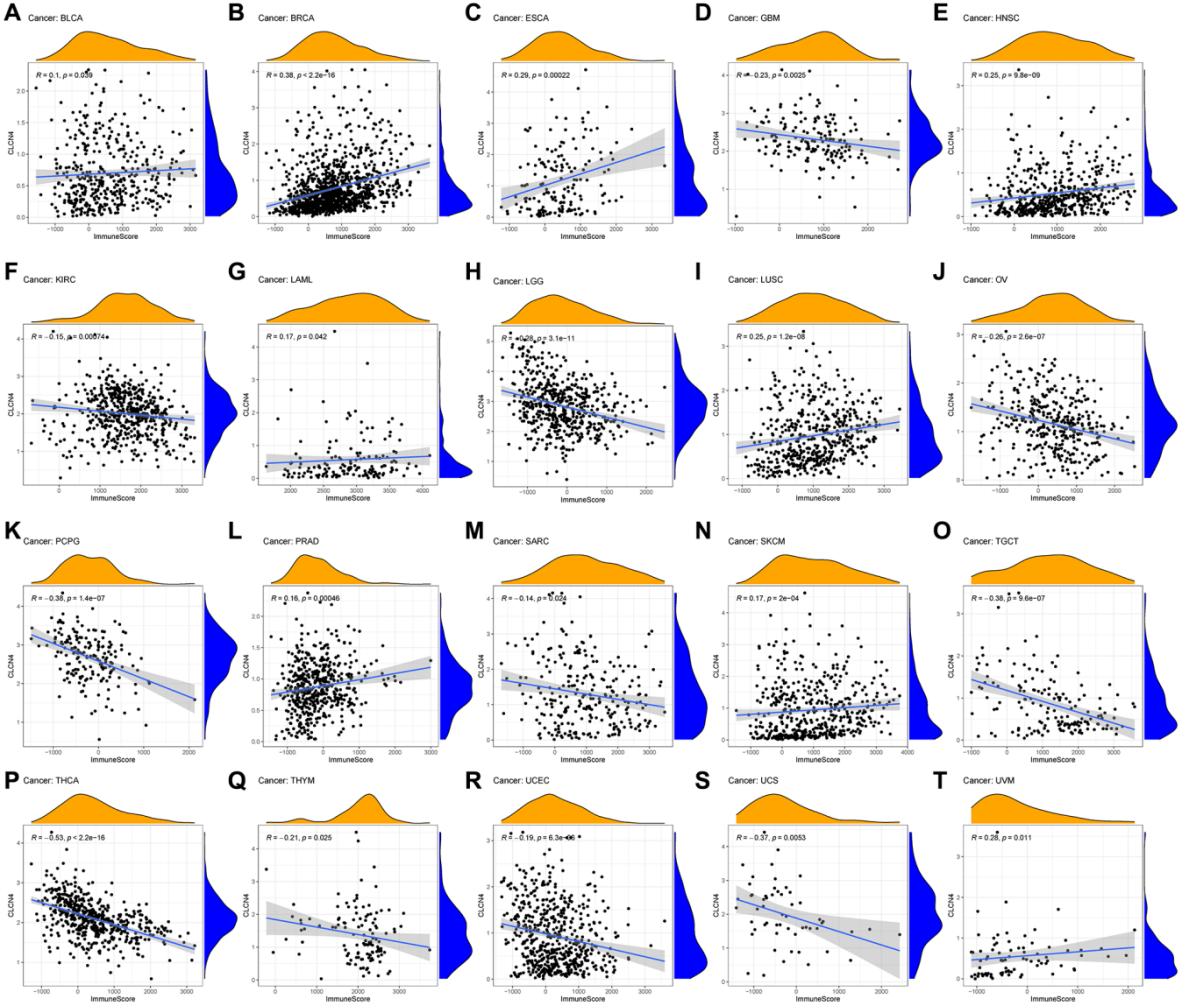
**Relationship between CLCN4 expression and Immune-Score.** (**A**) BLCA. (**B**) BRCA. (**C**) ESCA. (**D**) GBM. (**E**) HNSC. (**F**) KIRC. (**G**) LAML. (**H**) LGG. (**I**) LUSC. (**J**) OV. (**K**) PCPG. (**L**) PRAD. (**M**) SARC. (**N**) SKCM. (**O**) TGCT. (**P**) THCA. (**Q**) THYM. (**R**) UCEC. (**S**) UCS. (**T**) UVM.

To evaluate the connection between CLCN4 expression and tumor microenvironment, Immune-score and Stromal-score were combined. The findings showed that in the following tumor types: GBM, KIRC, LGG, OV, PCPG, SARC, TGCT, THCA, THYM, UCEC, and UCS, CLCN4 expression was adversely linked with the Immune-Score (Figure 6). In the LGG, OV, PCPG, THCA, UCEC, CLCN4 expression was negatively correlated with the Stromal-Score (Supplementary Figure 4).

### Correlation between CLCN4 and immune cells

**Figure 7:**
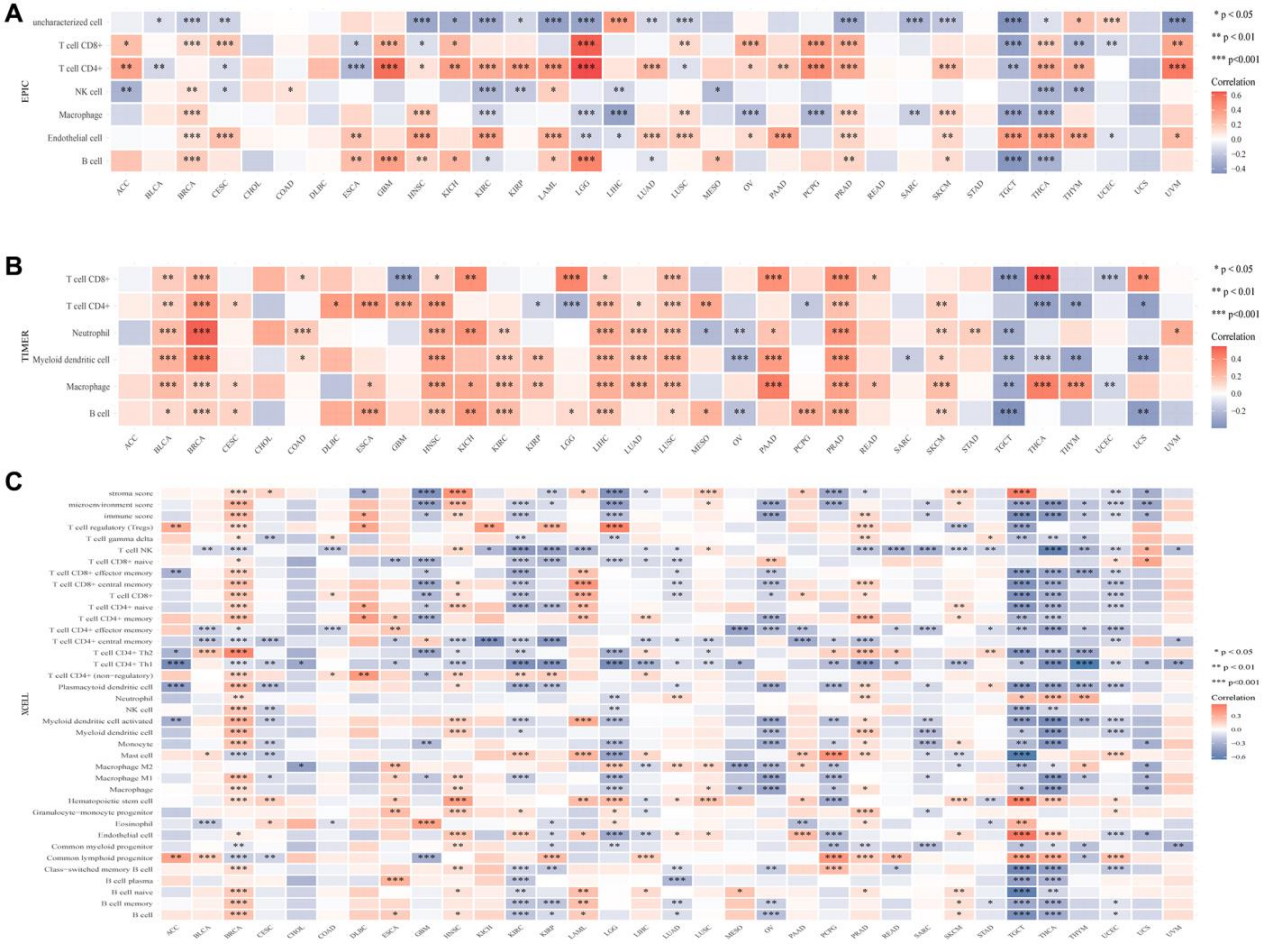
**The association between CLCN4 gene expression and 22 immune cells infiltration levels.** (**A**) The correlation between CLCN4 expression and immune cell infiltration was calculated according to EPIC algorithm. (**B**) The correlation between CLCN4 expression and immune cell infiltration was calculated according to the TIMER algorithm. (**C**) The correlation between CLCN4 expression and immune cell infiltration was calculated according to XCELL algorithm. ^*^p < 0.05, ^**^p < 0.01, ^***^p < 0.001.

The association between various immune cell infiltration levels and CLCN4 gene expression was investigated using the TIMER, CIBERSORT, EPIC, QUANTISEQ, XCELL, and MCPCOUNTER algorithms (Supplementary Figure 5). For the majority of cancer types, there was a significant correlation between CLCN4 expression and immune cell infiltration, especially CD4+ T cells. Based on EPIC algorithms, a statistical correlation was found between immune infiltration of CD4+ T cells and CLCN4 expression in 22 tumors. The results of the TIMER algorithm showed that CD4+ T cells was correlated with 19 tumors, and XCELL algorithms revealed a negative correlation between CD4+ Th1 cells and CLCN4 expression in 23 tumors (Figure 7).

### Correlation of CLCN4 with TMB and MSI

**Figure 8:**
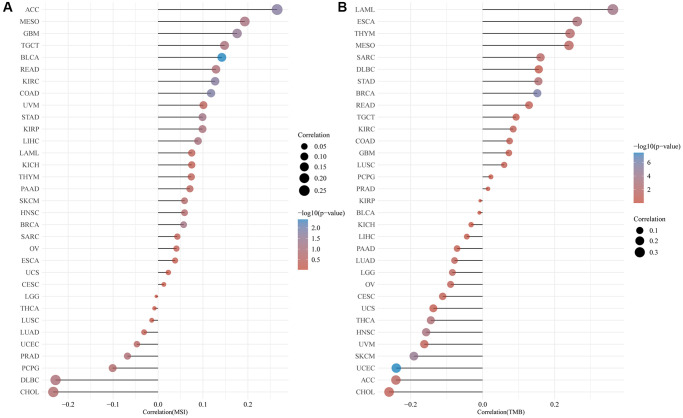
**Correlation of CLCN4 expression with microsatellite instability (MSI) and tumor mutational burden (TMB).** (**A**) Correlation between ClCN4 and MSI. (**B**) Correlation between CLCN4 and TMB.

Numerous studies have demonstrated the importance of MSI and TMB as indicators of tumor mutations in cancer cells. Using Spearman rank correlation coefficients, we calculated each tumor sample’s TMB, and examined the relationship between gene expression and TMB. There was a significant positive correlation between CLCN4 expression and TMB in LAML (*P* = 1.54E-04), ESCA (*P* = 1.05E-03), THYM (*P* = 8.39E-04), MESO (*P* = 3.12E-02), SARC (*P* = 1.5E-02), STAD (*P* = 2.87E-03), BRCA (*P* = 1.35E-06). However, it was negatively correlated with ACC (*P* = 3.39E-02), THCA (*P* = 1.55E-03), HNSC (*P* = 4.84E-04), SKCM (*P* = 3.46E-05) and UCEC (*P* = 3.92E-08). Using the same method, we analyzed the correlation between CLCN4 expression and MSI. We found: CLCN4 expression level was significantly positively correlated with GBM (*P* = 3.1E-02), KIRC (*P* = 1.92E-02), ACC (*P* = 1.81E-02), COAD (*P* = 1.44E-02), and BLCA (*P* = 4.05E-03). (Figure 8).

### CLCN4 is associated with the gene of MMR, DNA methyltransferases, copper death, immunostimulant, and chemokine

**Figure 9:**
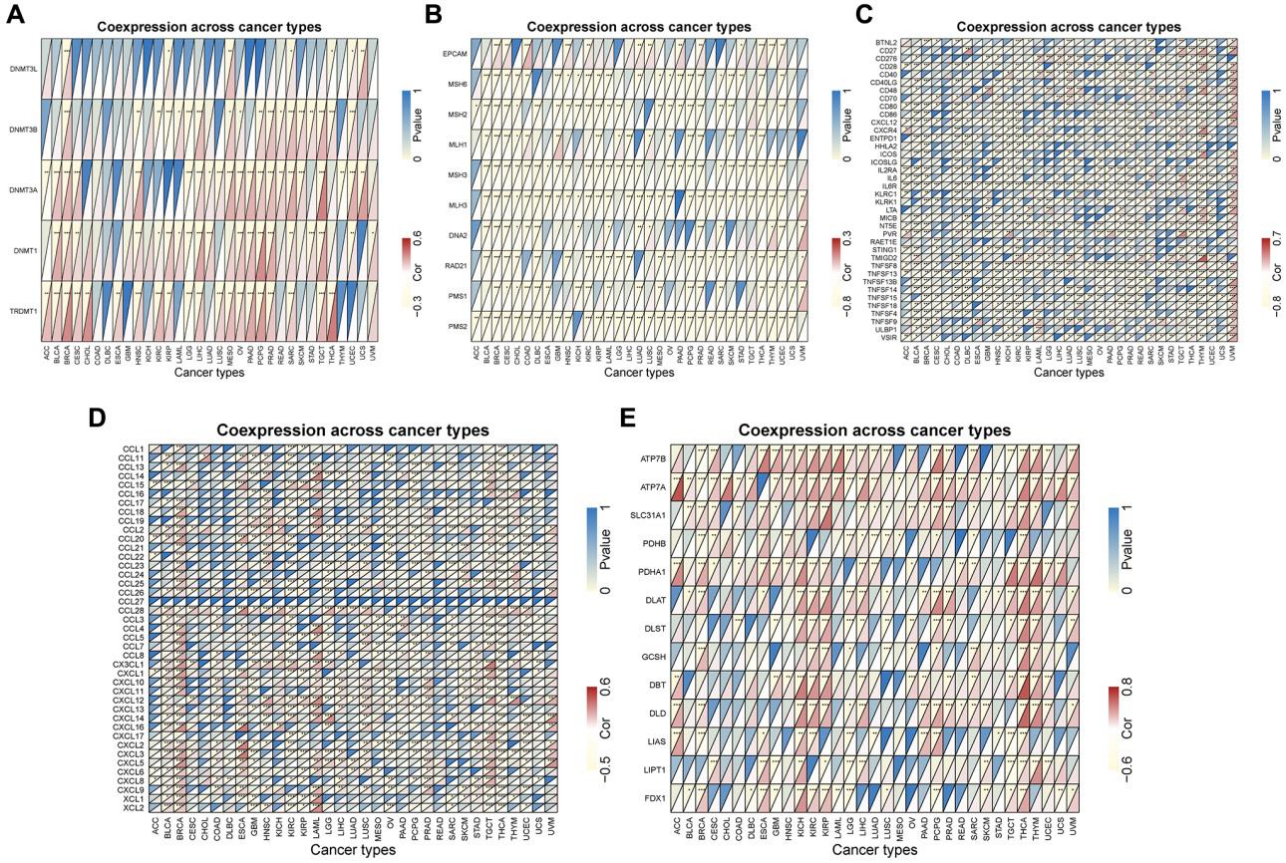
**Co-expression between CLCN4 expression and certain genes.** (**A**) DNA methyltransferase genes. (**B**) MMR genes. (**C**) immunostimulatory genes. (**D**) chemokine genes. (**E**) copper death gene. ^*^p < 0.05, ^**^p < 0.01, ^***^p < 0.001.

We evaluated the relationship between CLCN4 expression levels and mutation levels in the 10 MMR genes to ascertain CLCN4’s potential role in tumor progression. According to the findings in Figure 9B, the PMS2 had a significantly negatively correlated with the expression of CLCN4 in 31 tumors. The connections between CLCN4 and the five DNA methyltransferases were assessed (Figure 9A), DNMT3A had significantly negatively correlation in 21 cancers. As to the genes of copper death (Figure 9E), ATP7A is positively related to 29 cancers, while LIAS is negatively related to 13 tumors. According to the immunostimulatory (Figure 9C) and chemokine genes (Figure 9D), CXCL12 is positively correlated with 20 cancers and IL6R is negatively correlated with 29 tumors.

### CLCN4 expression in UCEC

**Figure 10:**
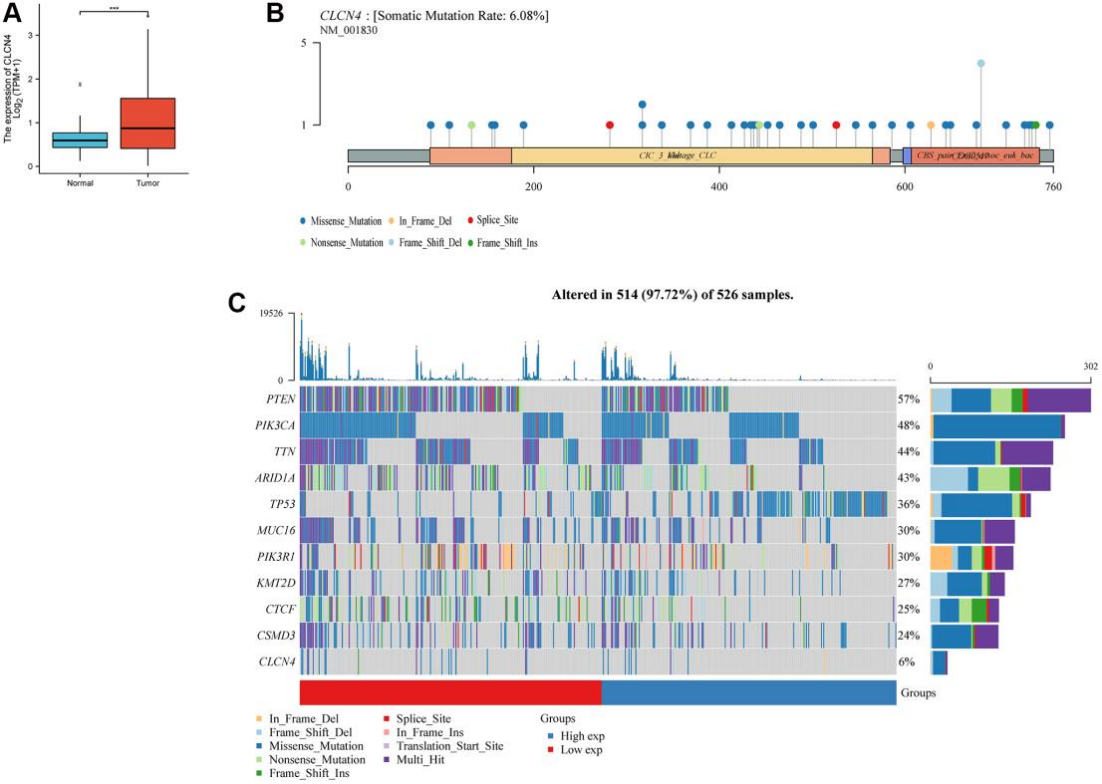
**The expression and mutation of CLCN4.** (**A**) Differential expression of CLCN4 in endometrial cancer and normal tissues (^***^p < 0.001). (**B**) The mutation of CLCN4 and the distribution of protein domains in the labeled hot lollipop graph. (**C**) Waterfall charts show different somatic mutation of several cancers in the CLCN4 high and low expression group.

According to the results discussed above, UCEC tumors, in particular, were at risk due to elevated expression of CLCN4. From the TCGA and GTEx databases, transcriptional data and clinical information about UCEC were retrieved for future investigation. CLCN4 was substantially expressed in UCEC tumor tissues when compared to normal tissues (*p* < 0.01). (Figure 10A).

### Increased expression of CLCN4 in tumor tissues of UCEC patients

**Figure 11:**
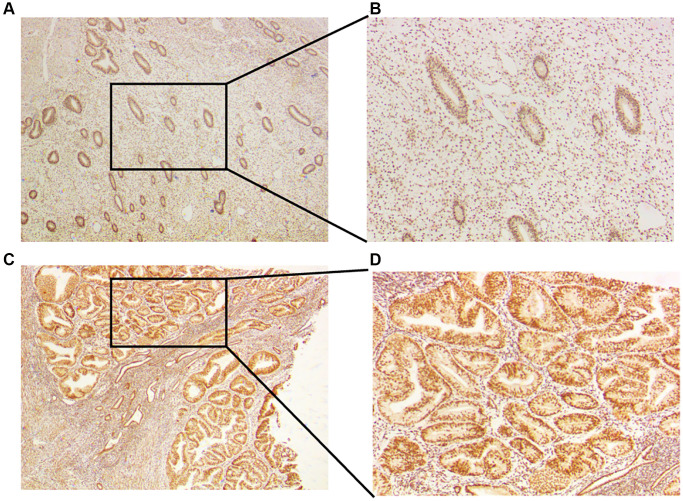
**Expression of CLCN4 in the UCEC patients.** (**A**, **B**) Immunohistochemical analysis of CLCN4 expression in normal endometrial tissues, an original magnification 40x, B original magnification 100x. (**C**, **D**) Immunohistochemical analysis of CLCN4 expression in the tumor tissues of UCEC patients, C original magnification 40x, D original magnification 100x.

To verify the expression of CLCN4 in UCEC, we treated tumor and normal tissues with IHC and found that CLCN4 were highly expressed in UCEC (Figure 11).

### Mutation landscape of CLCN4

The CLCN4 high and low expression groups’ somatic mutations were compared using R’s “maftools” package. Two related sets of prosperous scenarios are shown by waterfall charts. PTEN (57%), PIK3CA (48%), TIN (44%), and CLCN4 (6%), together with other genes with the highest mutation rates in the two groups, are displayed in the tumor’s abscissa in (Figure 10C). The labeled hot lollipop graph in (Figure 10B) displays the CLCN4 mutation as well as the distribution of protein domains.

### UCEC patients have a dismal prognosis when CLCN4 is present

**Figure 12:**
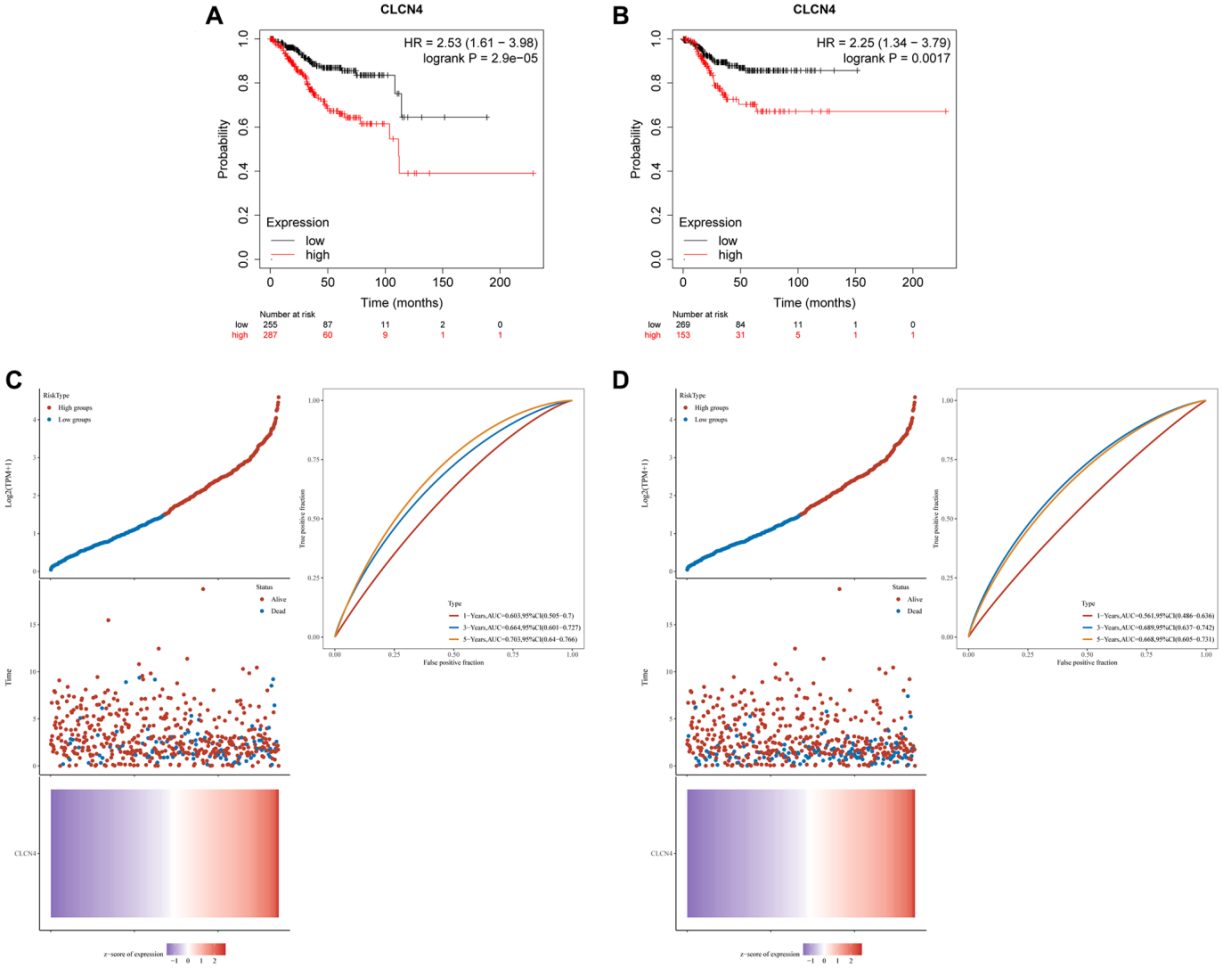
**The prognostic role of CLCN4 in UCEC.** (**A**) Kaplan–Meier analysis of the OS between the tumor and normal tissues. (**B**) Kaplan–Meier analysis of the RFS between the two groups. (**C**, **D**) Ranked dot and scatter plots showing the CLCN4 distribution and patient survival status. Heatmaps show CLCN4 Expression patterns of CLCN4. ROC curves to predict the sensitivity and specificity of 1-, 3-, and 5-year survival according to the CLCN4.

We assessed the prognostic value of CLCN4 in UCEC based on Affymetrix microarrays using the Kaplan-Meier plotter database, and constructed prognosis models for OS and PFS respectively. The findings indicate that a poor prognosis for UCEC was linked to increased expression of CLCN4. It was further established that CLCN4 expression was strongly linked with a poor prognosis of UCEC (OS *P* = 0.000029, PFS *P* = 0.0017) (Figure 12A, 12B). ROC curves to predict the sensitivity and specificity of 1-, 3-, and 5-year survival according to the CLCN4 expression. Following the calculation of each patient’s risk score, we used a heat map of CLCN4 and the “SurvMiner” R package to categorize patients into high-risk and low-risk groups at a median cutoff. (Figure 12C, 12D).

### Analysis of co-expression and role of enrichment

**Figure 13:**
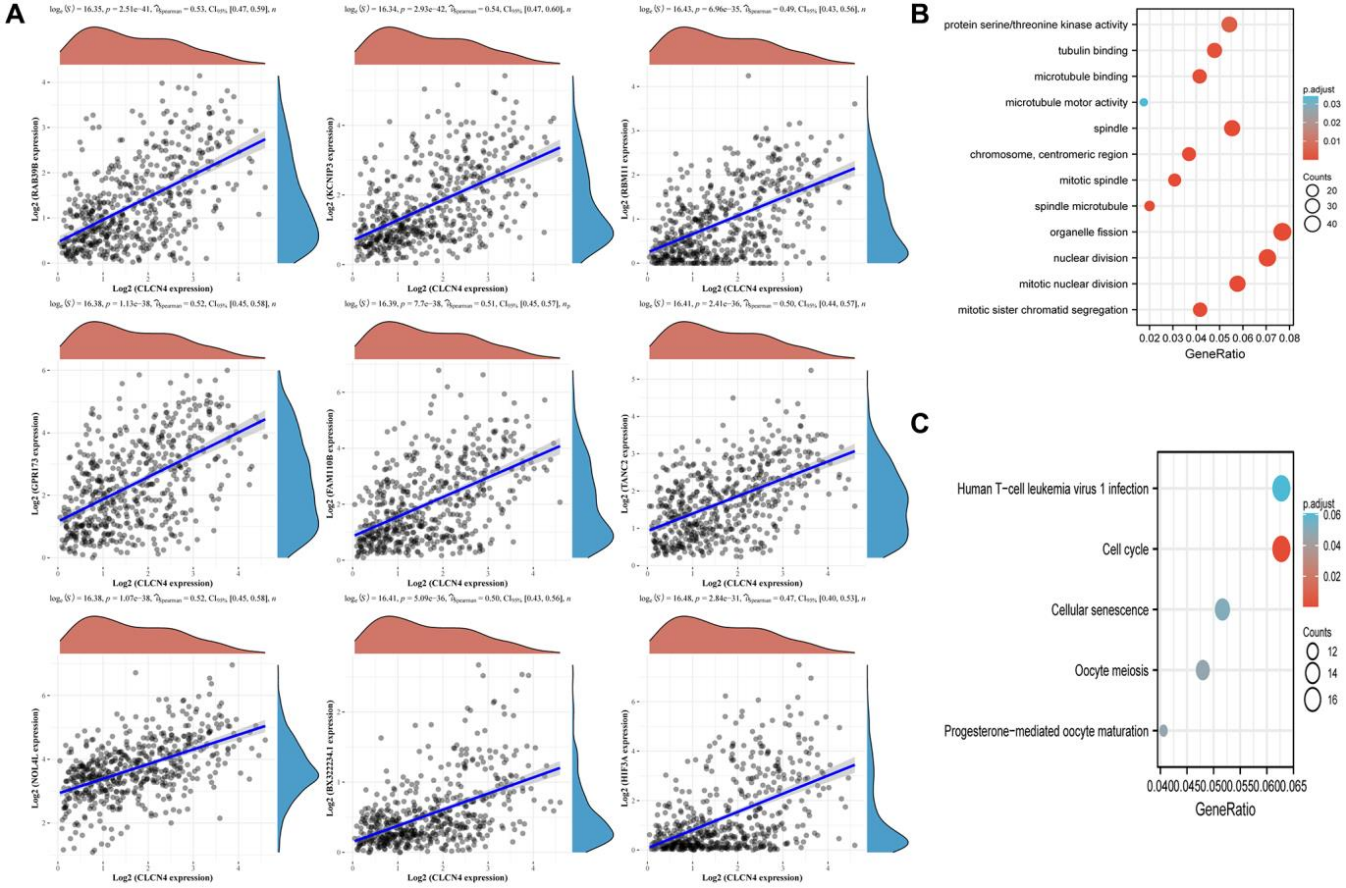
**Associated analysis of co-express genes.** (**A**) Relationship between CLCN4 and co-expression gene. (**B**) GO analysis of co-expressed genes. (**C**) Co-expressed gene KEGG analysis.

As shown in the (Figure 13A), which displays the top 9 genes most closely connected to CLCN4 expression, we performed a CLCN4 co-expression study to better understand the probable mechanism of CLCN4 in UCEC. The top 9 genes were RAB39B, KCNIP3, RBM11, GPR173, FAM110B, TANC2, NOL4L, BX322234.1, HIF3A. The top 400 co-expressed genes were then subjected to GO/KEGG analysis (Figure 13B, 13C), and the results revealed that BP was enriched for processes including mitotic nuclear division and organelle fission. The centromeric region, chromosome, and mitotic spindle were enriched in CC. Microtubule binding, protein serine/threonine kinase activity were enhanced in MF. Human T-cell Leukemia Virus 1 Infection and Cell Cycle were enriched, performed by KEGG Enrichment Analysis.

### CLCN4 knockdown inhibits UCEC cells migration and tumor invasion

**Figure 14:**
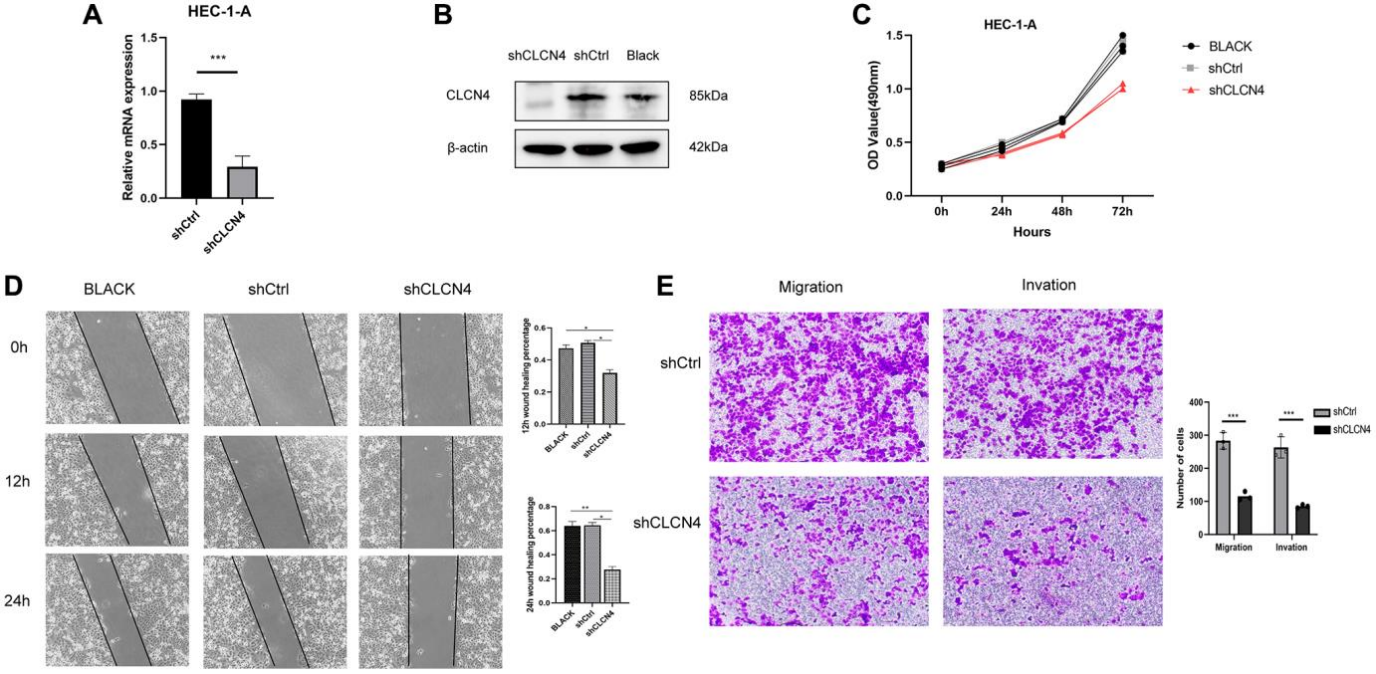
**Knockdown of CLCN4 inhabit the proliferation of HEC-1-A cells.** (**A**, **B**) qPCR and western blotting were used to verify the knockdown efficiency of shCLCN4. ^***^p < 0.001 (**C**) Growth curve was used to measure the effect of shCLCN4 on the proliferation of HEC-1-A cells by CCK8. (**D**) Wound healing of HEC-1-A cells between the BLCAK, shCtrl, shCLCN4 groups. ^*^p < 0.05, ^**^p < 0.01, ^***^p < 0.001. (**E**) Cell migration and migration of HEC-1-A cells between the shCtrl, shCLCN4 groups. ^***^p < 0.001.

The HEC-1-A cell line was cultured in our laboratory to verify the function of CLCN4 in UCEC. Our study showed that the knockdown efficiency of shCLCN4 was 64.7% (Figure 14A, 14B), and the knockdown of CLCN4 inhibited HEC-1-A cells proliferation (Figure 14C, *P* < 0.01). This study investigated the migration potential of UCEC cells by using wound healing and transwell assays. HEC-1-A cells migrating with CLCN4 knockdown were repressed (Figure 14D, *P* < 0.01). Matrigel transwell assays showed a significant reduction in HEC-1-A invasive potential when CLCN4 was knocked down. (Figure 14E, *P* < 0.001).

## DISCUSSION

The mortality rate associated with endometrial cancer has increased by 1.9% per year on average over 1971–2014 [30]. The incidence of UCEC was 417336 worldwide in 2020 [1]. 70–75% of endometrial cancer cases are diagnosed at FIGO stages I or II, and the five-year survival rate is between 75–90% [31], however, the five-year survival rate for stages III/IV is only 55–65% and 20–25%, respectively [31]. So, timely diagnosis is likely to have benefits for women in terms of improved survival, earlier-stage diagnosis and improved quality of life [32]. Typically, UCEC manifests as postmenopausal bleeding at an early stage, although only 5–10% of women with such bleeding have an ominous underlying condition [3]. Postmenopausal bleeding caused by endometrial cancer is less than 1% in women younger than 50, 3% in those over 55, and 24 percent in those older than 80 [33]. Obviously, abnormal bleeding is not an effective screening way for endometrial cancer. Identification and characterization of biomarkers is necessary to provide insights into molecular pathways of the disease and develop novel molecular-targeted therapies, for diagnosing early and estimating prognosis with greater specificity, particularly in recurrent and unfavorable disease outcomes [34].

Each year, more and more studies are conducted on biomarkers for endometrial cancer. PD-L1 expression in the tumor microenvironment may serve as a biomarker to identify patients who are likely to benefit from immunotherapy. Researchers have found that anti-PD-1 therapy is effective in PD-L1(+) tumors with response rates ranging from 36–100% compared with 0–17% in PD-L1-negative tumors [35]. Endometrial cancers exhibit 75% expression of PD-1, which is the highest among other gynecological cancers [36]. Pembrolizumab, a PD-L1 inhibitor approved by the FDA, is indicated for the treatment of unresectable or metastatic MSI or MMR solid tumors, including endometrial carcinomas [37]. IDO1 is a type of tryptophan catabolic enzyme [38], known for inactivating T cells and promoting tumor immunotolerance [39]. The IDO1 gene is high expressed in manly tumor cells included endometrial cancer [40]. IDO1 expression is also correlated with PD-L1, as most PD-L1(+) tumors show IDO1 expression [41], suggesting a synergistic effect on immunotherapy outcomes. Cyclooxygenase-2 (COX-2) is a rate-limiting enzyme, known as converting arachidonic acid into prostaglandins [42]. In normal tissues, COX-2 production is low [43], but it can be elevated during inflammation or during cancer formation [44]. In endometrial cancer, higher COX-2 levels are associated with frequent extrauterine involvement, shorter DFS (Disease free survival), higher grades, poor differentiation [45, 46]. For immune evasion, COX-2 expression is inversely related to CD8+T cell infiltration [47, 48].

Ion homeostasis of intracellular organelles is important for basic physiological activity such as ligand–receptor interactions, transport of neurotransmitters, transmembrane voltage. The acidification is caused by proton pumping activity of ATPases which need to be neutralized by a counter current, which is carried primarily by chloride ions [49, 50]. The chloride ions were previously thought to be mediated by Cl^−^ channels [51–53]. There are several pathologies associated with them, including impaired renal endocytosis and kidney stones (ClC-5) [52, 54], severe neurodegeneration (ClC-3) [55], handicapping intellectual development and epilepsy (ClC-4) [56–58], and reduced lysosomal storage (ClC-6) [59], osteopetrosis associated with neurodegeneration (ClC-7) [60]. CLC-4 is extensively expressed in the brain, heart, liver, kidney, and intestine [21, 22], CLCN4 mutations are associated with X-linked ID, epilepsy, behavior disorders, and dysmorphic features [56–58, 61]. To our knowledge, although, Prof. Soroceanu in 1999 made the first assertion that inhibiting chloride channels with drugs reduced the movement and infiltration of glioma cells in fetal rat brain tissue [23]. In 2010, Dr. T. Ishiguro identified CLCN4 as a promoter of colon cancer migration, invasion, and metastasis [26]. It is likely that colon cancer metastases to the liver are associated with elevated CLCN4 expression [26].

According to our findings, CLCN4 was highly expressed in 20 tumor types, such as ACC, CHOL, COAD, UCEC, etc. Higher CLCN4 levels were associated with poor OS (MESO, UCEC), short DSS (BLCA, MESO, and UCEC), low PFS (BLCA, UCEC), bad DFS (THCA, UCEC), Interestingly, across all four categories of prognostic factors, CLCN4 represents an obvious risk factor for UCEC, so we choose the endometrial cancer for in-depth analysis. It showed that CLCN4 overexpression was strongly linked with a poor prognosis of UCEC (OS *P* = 0.000029, PFS *P* = 0.0017).

Our results indicated that downregulation of CLCN4 exerted an inhibitory effect on endometrial tumor proliferation and migration. There are several possible mechanisms to explain why elevated CLCN4 promote cancer cells migration, invasion, and metastasis. Due to CLCN4’s role in ion exchange, the most likely mechanism is that it is a Cl-/proton antiporter [25]. The regulation of intracellular pH is crucial in tumor cells where proton accumulation is high and proton extrusion mechanism is essential for maintaining intracellular pH [62]. Furthermore, CLCN4 might promote invasiveness by modulating the pH of the endosomal compartment, for a large intracellular endosomal vesicle’s acidification promotes phagocytosed extracellular matrix degradation [63]. Another possibility is modulating salt levels in cells resulting from chloride fluxes, which could lead to water movement in and out, in this way, the cell shape can be altered to facilitate migration [64].

We were the first to explore the role of CLCN4 in the immune infiltration of UCEC cells. In our findings, The CLCN4 expression and CD4+T cells were tightly connected. According to XCELL algorithms, there was a negative correlation between the immune infiltration of T cell CD4+ Th1 and the expression of CLCN4 in 23 tumors. The negative connection between CLCN4 and T cell CD4+ Th1 in most cancers shows that CLCN4 may have a potential immunological mechanism. Professors Ikeda, Old, and Schreiber first proposed the “Cancer Immunoediting” model in 2002 [65, 66], describing immune system development in three distinct phases: elimination, equilibrium, and escape. At the elimination phase, endometrial cancer cells display “altered self” phenotypes and express “non-self” antigens [67], which are phagocytosed by dendritic cells (DCs) [68]. DCs are primed and then present these tumor-associated antigens to generate T cell responses including the production of CD8+ cytotoxic T cells (CTLs) and CD4+ T cells [68, 69]. A CD8+ CTL can directly kill EC cells, while a CD4+ helper cell can elicit a humoral or cytotoxic immune response [70]. In March 2020, for the first time, the national comprehensive cancer network (NCCN) recommended TCGA molecular typing in its endometrial cancer guidelines [71], indicating the dawn of immunotherapy based on tumor genotype and microenvironment. It is becoming clearer that endometrial cancer tissue is enriched with immune cells and cytokines, which can stimulate endogenous anti-tumor immunity [72, 73]. To our knowledge, although, there is no reports on the relationship between the immune infiltration and CLCN4 alterations in cancer development. But our results revealed that there was a negative correlation between the immune infiltration of T cell CD4+ Th1 and the CLCN4 expression. 1. We speculate that CLCN4 may have a role on the CD4+ T cells to altered immune infiltration.

We should be conscious of the limitations of our research. First off, bioinformatics techniques were used to analyze data from the public databases. It is necessary to perform *in vivo* experiments to determine the mechanism through which CLCN4 promotes tumor growth. The results need to be confirmed in both basic and clinical research.

## Supplementary Materials

Supplementary Figures
