# Supplementary material for: Comprehensive analysis and experimental validation reveal elevated CLCN4 is a promising biomarker in endometrial cancer
Source: Aging (Albany NY). 2023 Sep 5;15(17):8744–69. doi: 10.18632/aging.204994 (PMC10522378; doi:10.18632/aging.204994)
Supplement: Supplementary Figures [file aging-15-204994-s001.pdf]

SUPPLEMENTARY FIGURES

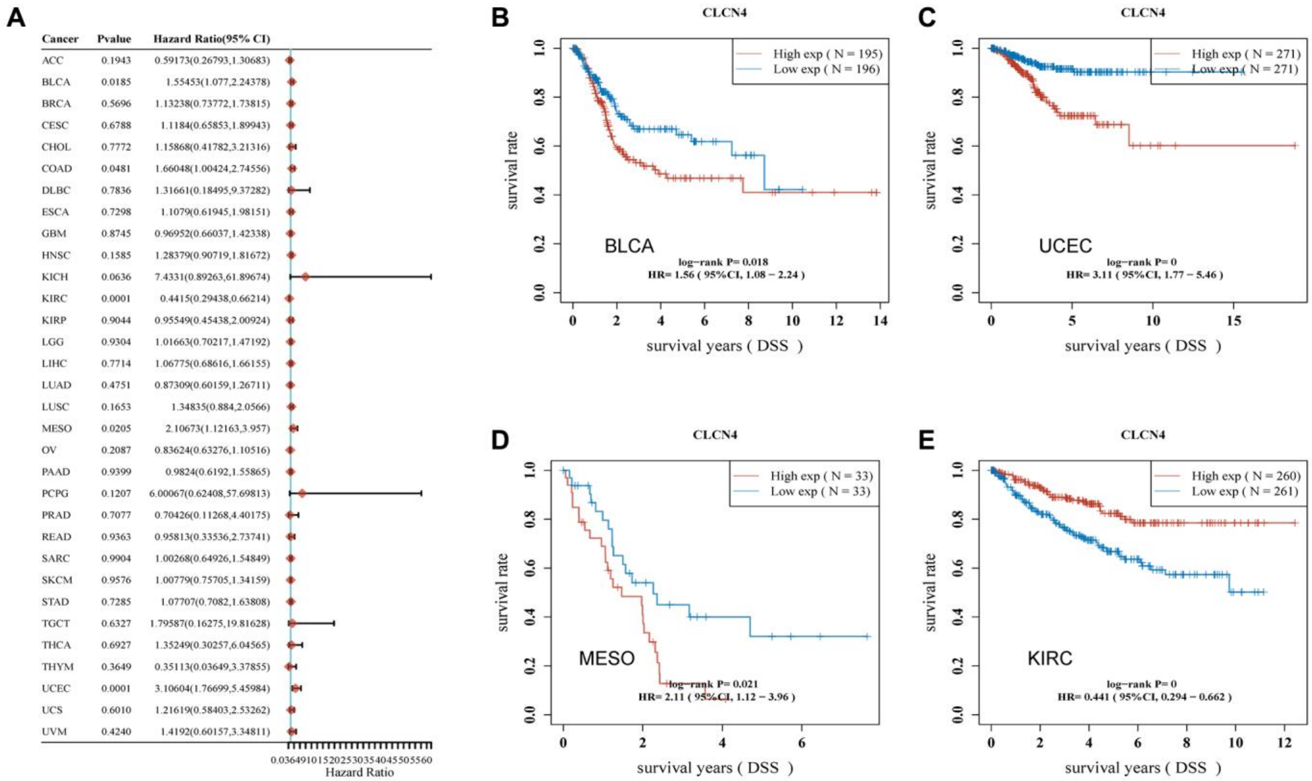

Supplementary Figure 1. Association of CLCN4 with DSS in pan-cancer. (**A**) The correlation between CLCN4 expression and DSS in various tumors used cox regression model. (**B**–**E**) DSS Kaplan-Meier curve: (**B**) BLCA, (**C**) UCEC, (**D**) MESO, (**E**) KIRC.

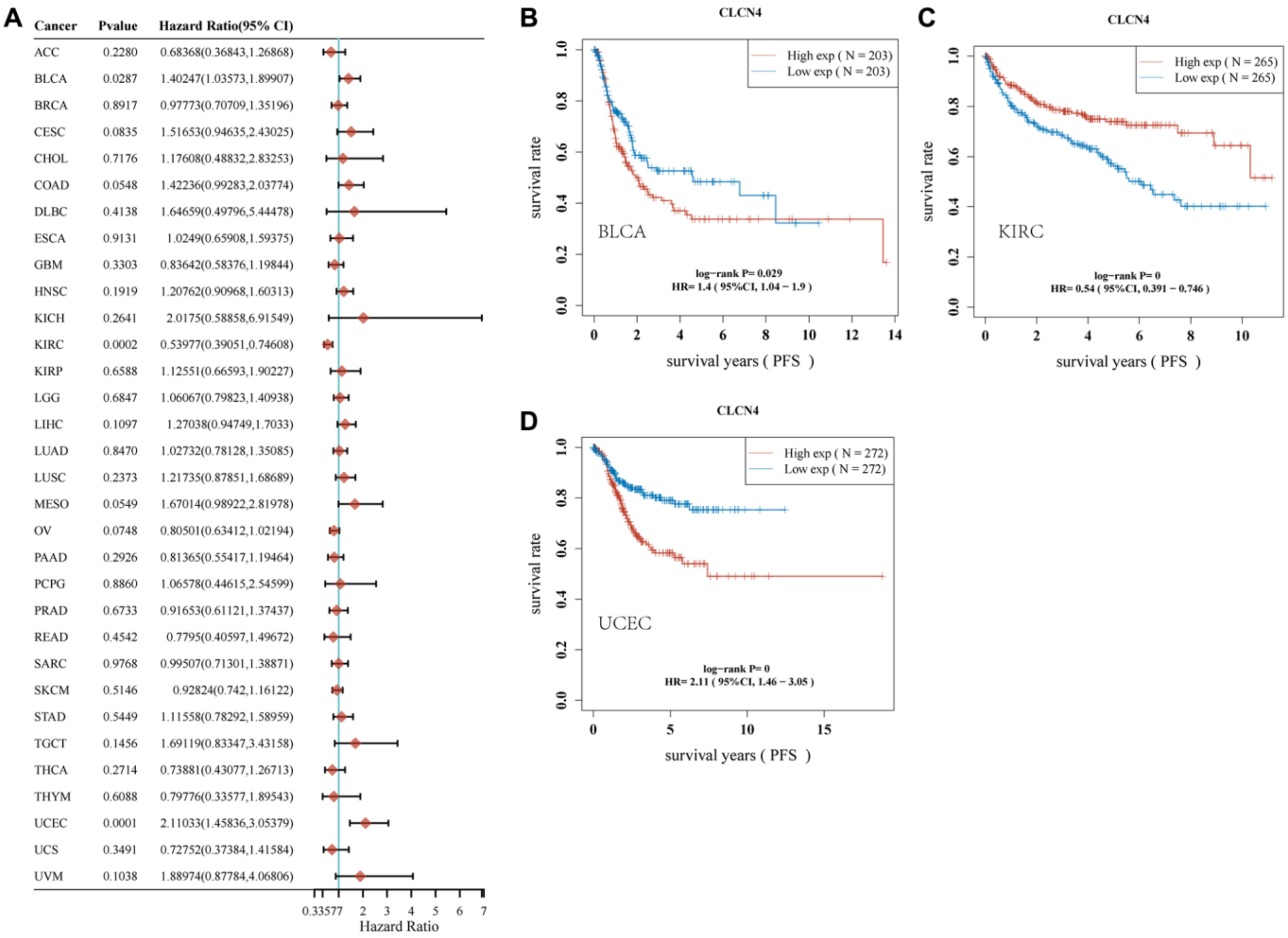

Supplementary Figure 2. Association of CLCN4 with PFS in pan-cancer. (**A**) The correlation between CLCN4 expression and PFS in various tumors used cox regression model. (**B**–**D**) PFS Kaplan-Meier curve.

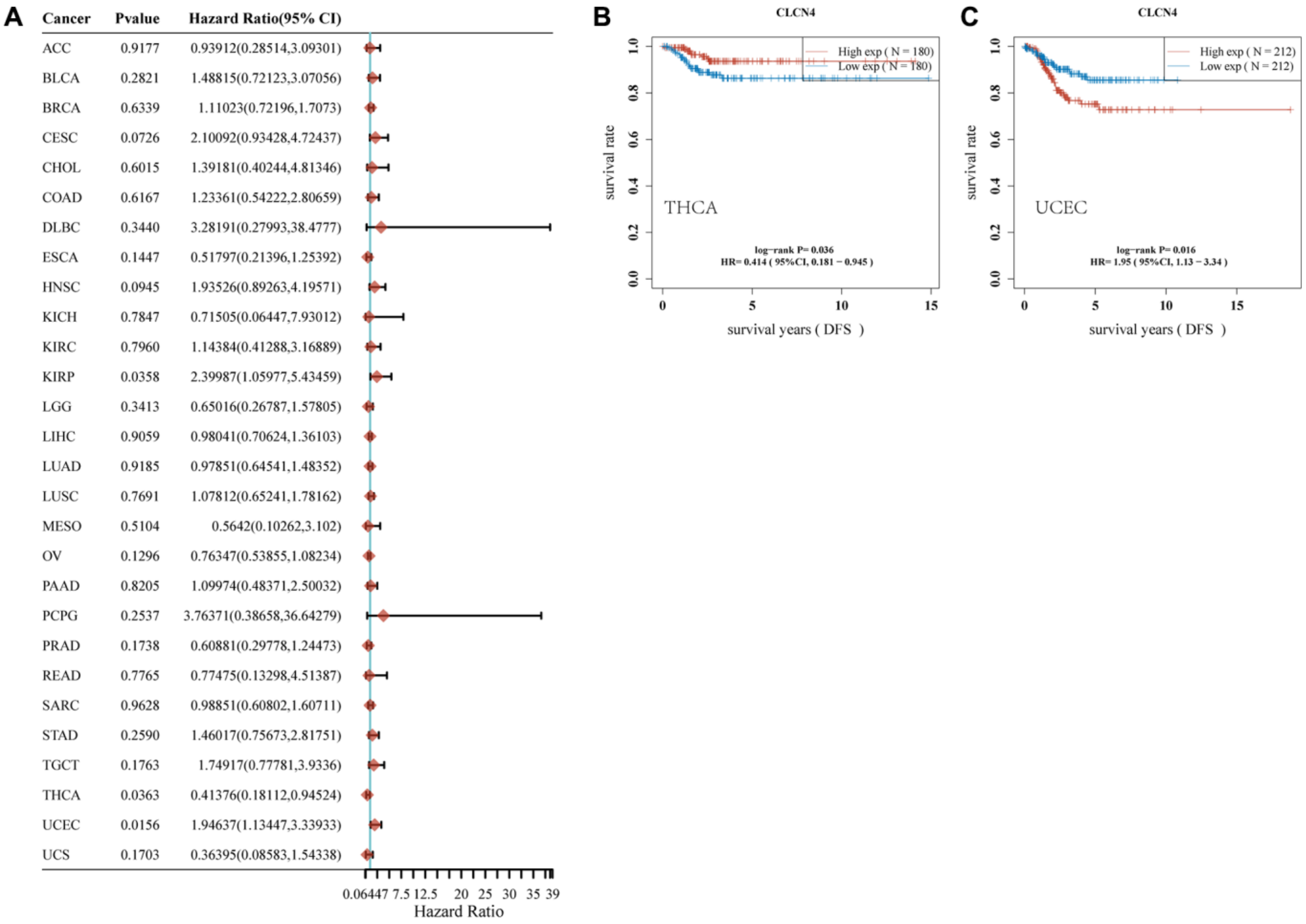

Supplementary Figure 3. Association of CLCN4 with DFS in pan-cancer. (**A**) The correlation between CLCN4 expression and DFS in various tumors used cox regression model. (**B**, **C**) DFS Kaplan-Meier curve.

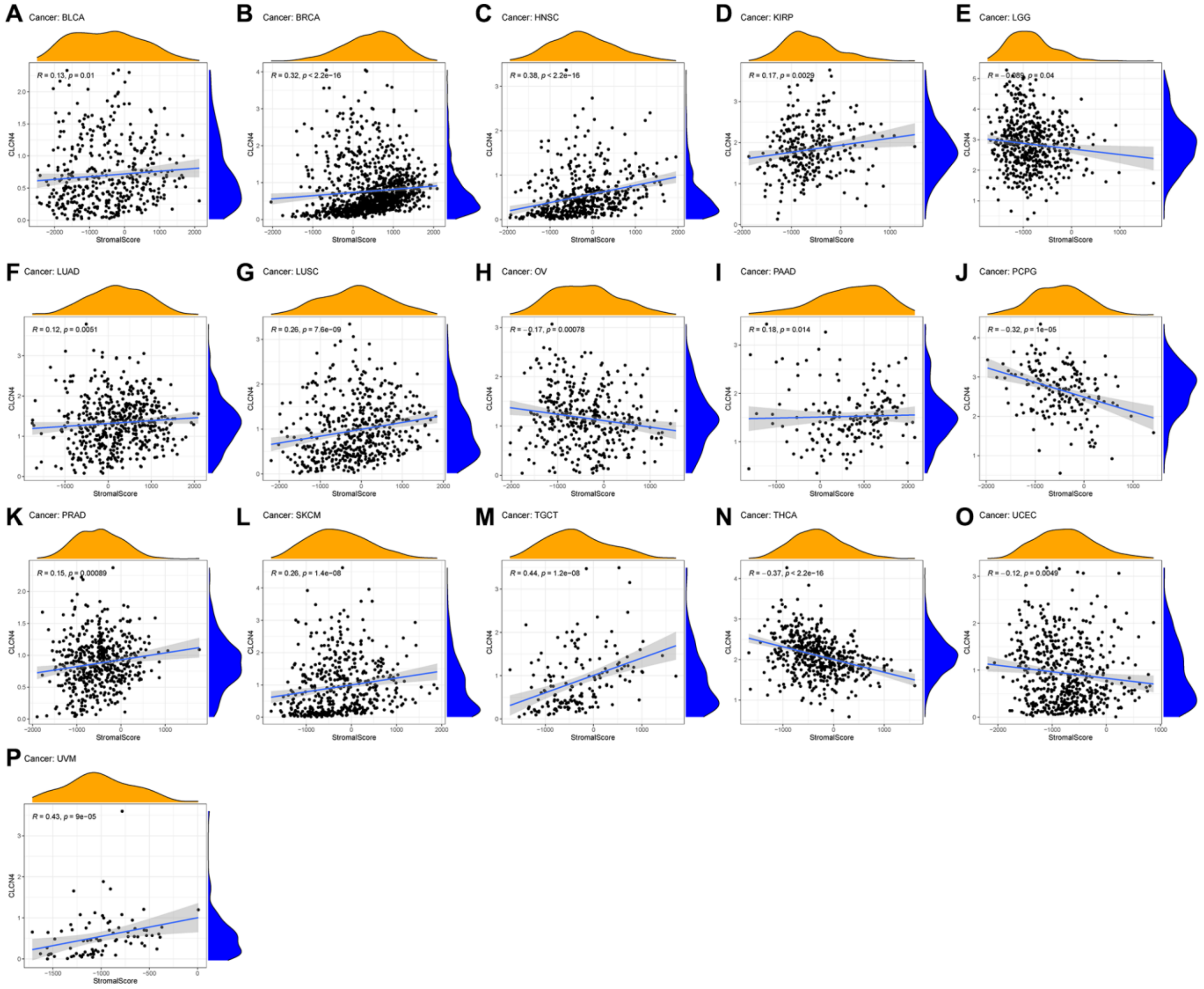

Supplementary Figure 4. Relationship between CLCN4 expression and Stromal-Score. (**A**) BLCA. (**B**) BRCA. (**C**) HNSC. (**D**) KIRP. (**E**) LGG. (**F**) LUAD. (**G**) LUSC. (**H**) OV. (**I**) PAAD. (**J**) PCPG. (**K**) PRAD. (**L**) SKCM. (**M**) TGCT. (**N**) THCA. (**O**) UCEC. (**P**) UVM.

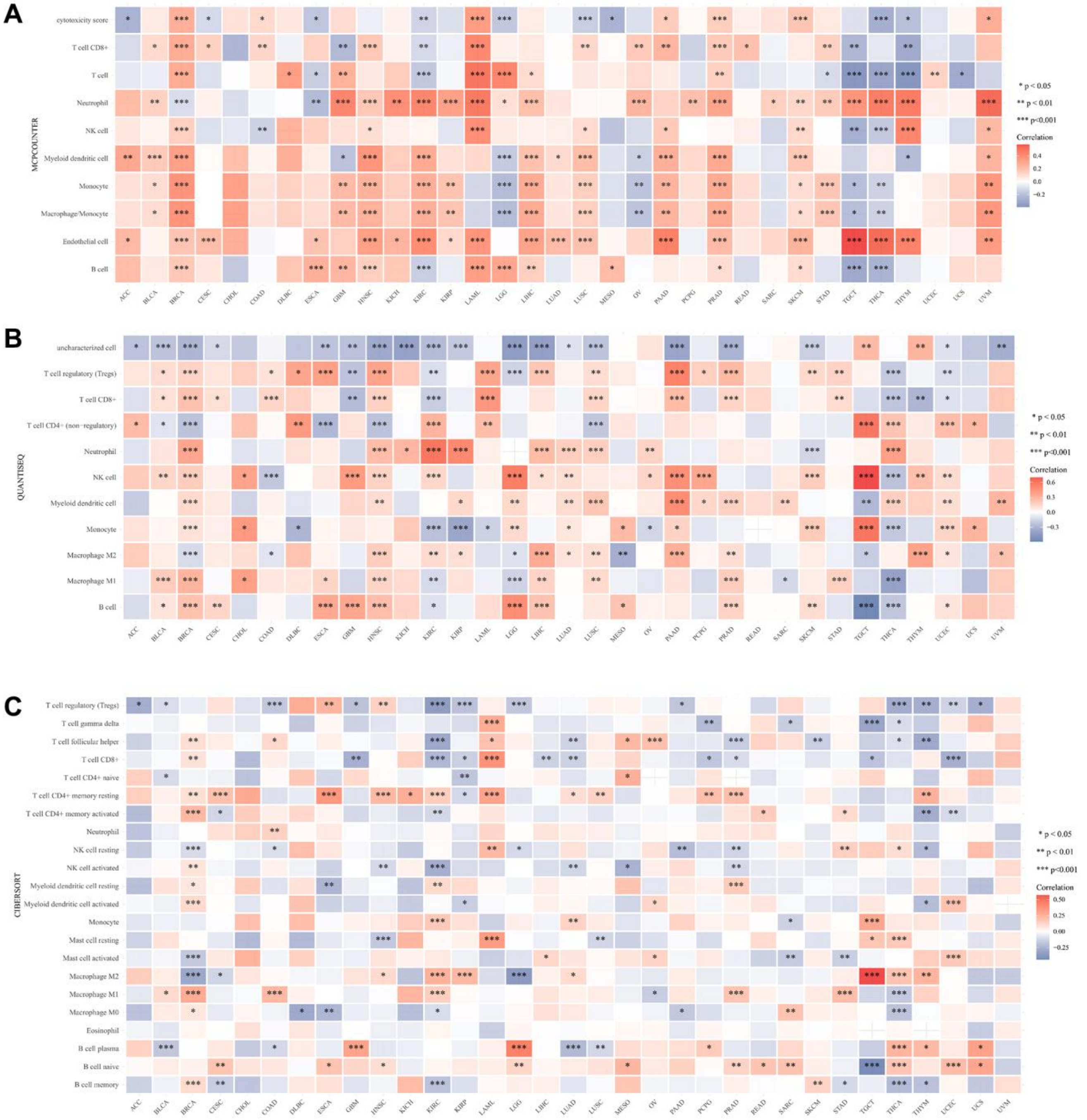

Supplementary Figure 5. The association between CLCN4 gene expression and 22 immune cells infiltration levels. (**A**) The correlation between CLCN4 expression and immune cell infiltration was calculated according to MCPCOUNTER algorithm. (**B**) The correlation between CLCN4 expression and immune cell infiltration was calculated according to the QUANTISEQ algorithm. (**C**) The correlation between CLCN4 expression and immune cell infiltration was calculated according to CIBERSORT algorithm. $^{*}p < 0.05$, $^{**}p < 0.01$, $^{***}p < 0.001$.
